# Supplementary material for: Curated and harmonised transcriptomics datasets of interstitial lung diseases
Source: Data Brief. 2025 Oct 14;63:112139. doi: 10.1016/j.dib.2025.112139 (PMC12581653; doi:10.1016/j.dib.2025.112139)
Supplement: Supplementary file 1 [file mmc1.zip › Supplementary_material/RNA-seq/fastQC reports/GSE169500/SRR14057106_fastqc.html]

SRR14057106.fastq.gz FastQC Report 

FastQC Report

Mon 5 Feb 2024  
SRR14057106.fastq.gz

## Summary

- Basic Statistics
- Per base sequence quality
- Per sequence quality scores
- Per base sequence content
- Per sequence GC content
- Per base N content
- Sequence Length Distribution
- Sequence Duplication Levels
- Overrepresented sequences
- Adapter Content

## Basic Statistics

| Measure | Value |
| --- | --- |
| Filename | SRR14057106.fastq.gz |
| File type | Conventional base calls |
| Encoding | Sanger / Illumina 1.9 |
| Total Sequences | 21760236 |
| Sequences flagged as poor quality | 0 |
| Sequence length | 8-372 |
| %GC | 50 |

## Per base sequence quality

## Per sequence quality scores

## Per base sequence content

## Per sequence GC content

## Per base N content

## Sequence Length Distribution

## Sequence Duplication Levels

## Overrepresented sequences

| Sequence | Count | Percentage | Possible Source |
| --- | --- | --- | --- |
| GGGCCACGAGCTGAGTGCGTCCTGTCACTCCACTCCCATGTCCCTTGGGA | 283158 | 1.3012634605617328 | No Hit |
| GCTGCTACAGGAGAATAGCAGACAGGTATAGTTAAGAAACAACTTTATTT | 111029 | 0.5102380323448698 | No Hit |
| ACACTGAGGACTCTGTTCCTCTCCTTTCCGCCTAGGGGAAAGTCCCCGGA | 85583 | 0.3932999623717316 | No Hit |
| AAAGCCAAGAAAACCAACGATGCCAGATACTGAATTTCTATAGCACTGAC | 67119 | 0.3084479414653407 | No Hit |
| TCTTTCTGGCCTGGAGGCTATCCAGCGTACTCCAAAGATTCAGGTTTACT | 51186 | 0.23522722823410552 | No Hit |
| AAGCTCAGGGAGAGCCTGTTAGGGCCGCCTCTGGCCTAGTCTCAGACCTT | 49797 | 0.22884402540487153 | No Hit |
| AAGACCAACGTCAAGGCCGCCTGGGGTAAGGTCGGCGCGCACGCTGGCGA | 46459 | 0.21350411824577636 | No Hit |
| AAGCTCAGGGAGAGCCTGTTAGGGCCGCCTCTGGCCCTAGTCTCAGACCT | 40439 | 0.18583897711403496 | No Hit |
| TCTGCAATGCAATCACAATGCCCAAACTAGACCTGCCATTTCTCACACAC | 39786 | 0.18283809054276803 | No Hit |
| CTAAGTGCTGTGTTGTCGTTCCCCCTGCTTAAAATAAAGTTGTTTCTTAA | 39614 | 0.182047657939004 | No Hit |
| TTTACACCTGCTAGGGTGTTCAAAGGTCAGTGCTATAGAAATTCAGTATC | 33529 | 0.15408380681165407 | No Hit |
| TCTGTCTTCTTCAGTTTCGACTTATCGAATTTCTCGATCTCAGCCATATC | 32725 | 0.1503889939428966 | No Hit |
| TCACACTTCATGATGGAGTTGAAGGTAGTTTCGTGGATGCCACAGGACTC | 30885 | 0.14193320329797893 | No Hit |
| ACATAGCAATTCAGGAAATTTGACTTTCCATTCTCTGCTGGATGACGTGA | 29917 | 0.13748472213260923 | No Hit |
| TACTCGTGCGCCTCGCTTTGCTTTTCCTCCGCAACCATGTCTGACAAACC | 26484 | 0.12170823882608627 | No Hit |
| AAGCTCAGGGAGAGCCTGTTAGGGCCGCTCTGGCCTAGTCTCAGACCTTC | 26336 | 0.12102809914377767 | No Hit |
| ATTCTTCCTACCCATGAGCATGGAATGTTCTTCCATTTGTTTGTATCCTC | 26284 | 0.12078913114729087 | No Hit |
| TGTTCCCTCTCCTCATGAGATTGGTGAAGAAAGTATTTGGCAAAGTTCTT | 25727 | 0.11822941626184569 | No Hit |
| TGACTGGTGATGCTGATATTGGAGTGCATCTGAGGCTGTTGTAGAAATAA | 25667 | 0.11795368395820707 | No Hit |
| TGGCTCTCCCAGAGGCAGAAGGACAATGGCTGTTTCAGGAGCTCTGGGTC | 25465 | 0.1170253852026237 | No Hit |
| AAAACAGCGTCTTACTGGGGAGGGATATGTGCTTTCTCATCTATCCTCAG | 25363 | 0.11655664028643808 | No Hit |
| TGTTGTGCTAAACGCTGGGAGGGTACAAGTTGGTCATTCCTAAATCTGTG | 23768 | 0.10922675654804478 | No Hit |
| GAAGACGTACCTTTGTTGGAAGACATACCTTTGTTGGAAGACGTACCTTT | 23279 | 0.10697953827339005 | No Hit |
| TGTCCATGTCTTACTACTTTGACCGCGATGATGTGGCTTTGAAGAACTTT | 22850 | 0.10500805230237392 | No Hit |
| ACACTGAGGACTCTGTTCCTCTCCTTCCGCCTAGGGGAAAGTCCCCGGAC | 22336 | 0.10264594556786977 | No Hit |
| AAACCAGACATGGGGGAAATCGCCAGCTTCGATAAGGCCAAGCTGAAGAA | 21997 | 0.10108805805231157 | No Hit |

## Adapter Content

Produced by FastQC (version 0.11.7)
